# Do running speed and shoe cushioning influence impact loading and tibial shock in basketball players?

**DOI:** 10.7717/peerj.4753

**Published:** 2018-05-11

**Authors:** Wing-Kai Lam, Jacobus Liebenberg, Jeonghyun Woo, Sang-Kyoon Park, Suk-Hoon Yoon, Roy Tsz-Hei Cheung, Jiseon Ryu

**Affiliations:** 1 Department of Kinesiology, Shenyang Sport University, Shenyang, China; 2 Li Ning Sports Sciences Research Center, Li Ning (China) Sports Goods Co., Ltd., Beijing, China; 3 Institute of General Kinesiology and Athletic Training, University of Leipzig, Leipzig, Germany; 4 Motion Innovation Centre, Korea National Sport University, Seoul, Korea; 5 Gait & Motion Analysis Laboratory, Department of Rehabilitation Sciences, Hong Kong Polytechnic University, Hong Kong, Hong Kong

**Keywords:** Footwear, Peak acceleration, Ground reaction force, Kinetics, Footstrike, Loading rate

## Abstract

**Background:**

Tibial stress fracture (TSF) is a common injury in basketball players. This condition has been associated with high tibial shock and impact loading, which can be affected by running speed, footwear condition, and footstrike pattern. However, these relationships were established in runners but not in basketball players, with very little research done on impact loading and speed. Hence, this study compared tibial shock, impact loading, and foot strike pattern in basketball players running at different speeds with different shoe cushioning properties/performances.

**Methods:**

Eighteen male collegiate basketball players performed straight running trials with different shoe cushioning (regular-, better-, and best-cushioning) and running speed conditions (3.0 m/s vs. 6.0 m/s) on a flat instrumented runway. Tri-axial accelerometer, force plate and motion capture system were used to determine tibial accelerations, vertical ground reaction forces and footstrike patterns in each condition, respectively. Comfort perception was indicated on a 150 mm Visual Analogue Scale. A 2 (speed) × 3 (footwear) repeated measures ANOVA was used to examine the main effects of shoe cushioning and running speeds.

**Results:**

Greater tibial shock (*P* < 0.001; *η*^2^ = 0.80) and impact loading (*P* < 0.001; *η*^2^ = 0.73–0.87) were experienced at faster running speeds. Interestingly, shoes with regular-cushioning or best-cushioning resulted in greater tibial shock (*P* = 0.03; *η*^2^ = 0.39) and impact loading (*P* = 0.03; *η*^2^ = 0.38–0.68) than shoes with better-cushioning. Basketball players continued using a rearfoot strike during running, regardless of running speed and footwear cushioning conditions (*P* > 0.14; η^2^ = 0.13).

**Discussion:**

There may be an optimal band of shoe cushioning for better protection against TSF. These findings may provide insights to formulate rehabilitation protocols for basketball players who are recovering from TSF.

## Introduction

Basketball is a popular sport with more than 450 million participants worldwide (International Basketball Federation, 2016), making it an important sport for injury prevention research in order to improve healthy living. Apart from jumping, cutting, and turning, running in a straight line is an indispensable and essential task during basketball games. On average, a basketball player performs 3.4 km of running at a pace of 4 m/s per game (Ben Abdelkrim, El Fazaa & El Ati, 2007). Running exercise is one of the key elements to improve conditioning attributes of basketball players such as general fitness (endurance runs), anaerobic endurance that overcomes fatigue (interval runs) and muscle endurance (resisted runs) (National Basketball Conditioning Coaches Association, 2007; Ben Abdelkrim et al., 2010). However, the biomechanics of running in basketball players remain unclear, especially characteristics related to impact loading. Additionally, most players wear the same basketball shoes for training on the court and in the fitness room. Thus, it is currently unknown to what extent basketball shoe cushioning influences impact loading during running in basketball players.

Tibial stress fracture (TSF) is one of the most common overuse injuries in collegiate basketball players, which accounts for 10 chronic injuries per 1,000 basketball games (Iwamoto & Takeda, 2003; Meeuwisse, Sellmer & Hagel, 2003). Although TSF has been extensively studied, the etiology of TSF has yet to be determined (Milgrom et al., 2015). Previous research suggested that TSF may relate to high level of tibial shock (Crowell & Davis, 2011), impact peak (Davis, Milner & Hamill, 2004), and vertical loading rates (Milner et al., 2006), in the running population. These kinetic parameters have been shown to be influenced by many factors in runners, including running speed (Edwards et al., 2010), cushioning performance of the running shoes (Miller & Hamill, 2009), and initial footstrike pattern (Chen et al., 2016). It has been proposed that slower running speeds and better cushioned shoes would lower the risk of TSF in runners (Edwards et al., 2010; Miller & Hamill, 2009). However, similar risk of TSF in runners with different footstrike patterns was reported in a recent computational study, despite the fact that significantly higher vertical loading rates were observed in runners with rearfoot strike compared to those with midfoot or forefoot strike (Chen et al., 2016).

However, findings from these studies involving the running population may not be directly applicable to basketball players, simply because of the difference between runners and basketball players in terms of their muscle development, physical training regimen, functional demand of the footwear design, and postural adjustment during gait (Leroy et al., 2000). Compared to distance runners, it has been suggested that athletes who require extensive power training (e.g., basketball players, Wissel, 2012) may demonstrate greater ankle stiffness (Hobara et al., 2008). This is due to the nature of how ankle stiffness is measured by looking at the kinematics and kinetics which will implicitly result in a change in biomechanics. In addition, most previous basketball studies focused on cutting or jump landing (Cong et al., 2014; Nin, Lam & Kong, 2016; Lam et al., 2015), with a small amount of research being done on running biomechanics, even though it is an essential component of a basketball game or training session (Ben Abdelkrim, El Fazaa & El Ati, 2007). Since basketball players might adapt differently to running speed and footwear cushioning when compared to runners, further analysis of specific aspects of the accelerometry and ground reaction force would need to be assessed in order to analyze impact characteristics in running. Some previous research has been done on basketball footwear where evaluated plantar loading was found when basketball players performed running and sprinting movements (Guettler et al., 2006; Yu et al., 2007).

Hence, this study compared the tibial shock, impact peak, vertical loading rate, and initial footstrike angle of basketball players running at different speeds wearing basketball shoes. Based on the previous findings in running research, it was hypothesized that slower running speeds or more cushioned basketball shoes would result in lower impact loading. Another hypothesis is that basketball players would land with a rearfoot landing pattern at slower running speeds, or when they put on basketball shoes with better cushioning performance (Breine et al., 2014).

## Materials and Methods

### Participants

Eighteen male basketball players (mean (SD) age = 25.0 (2.3) years; height = 179.0 (4.6) cm; mass = 74.4 (6.5) kg) were recruited for this study. All the participants had at least four years of competitive basketball experience and attended practice for more than 4 h per week. The study focused on collegiate basketball players because they represented a population closer to recreational basketball players which are more prone to injury than professional players (Meeuwisse, Sellmer & Hagel, 2003; Messina, Farney & DeLee, 1999). Participants were free from any injury six months before the experiment were conducted, and had received no prior lower extremity surgery up till the time of the study. Ethical approval was granted by Li Ning institutional review committee (IRB-2015BM007). All participants signed an informed consent form prior to the start of the study.

### Test shoe conditions

Three pairs of new basketball shoes were selected based on their cushioning performance in the standard mechanical impact attenuation test procedure (ASTM protocol F1976-13). This was done with a mechanical impact tester (Exeter Research V2.6; Exeter Research, Brentwood, NH, USA). Thirty consecutive mechanical impact trials were performed at the center of the heel region with an 8.5-kg mass dropping from a 50-mm height. The cushioning properties of a shoe were averaged with the last five trials. This standard assessment procedure allowed objective judgment for cross studies comparison (Nin, Lam & Kong, 2016; Sterzing, Lam & Cheung, 2012). The three test shoe models were classified as best-cushioning shoe (9.8 g-force), better-cushioning shoe (11.3 g-force), and regular-cushioning shoe (12.9 g-force), which represents the available range of impact scores among the available basketball shoes (ranged from 9.8 to 12.9 g-force) in the market. Shoes with lower impact score would indicate better shoe cushioning performance.

### Testing procedures

**Figure 1:**
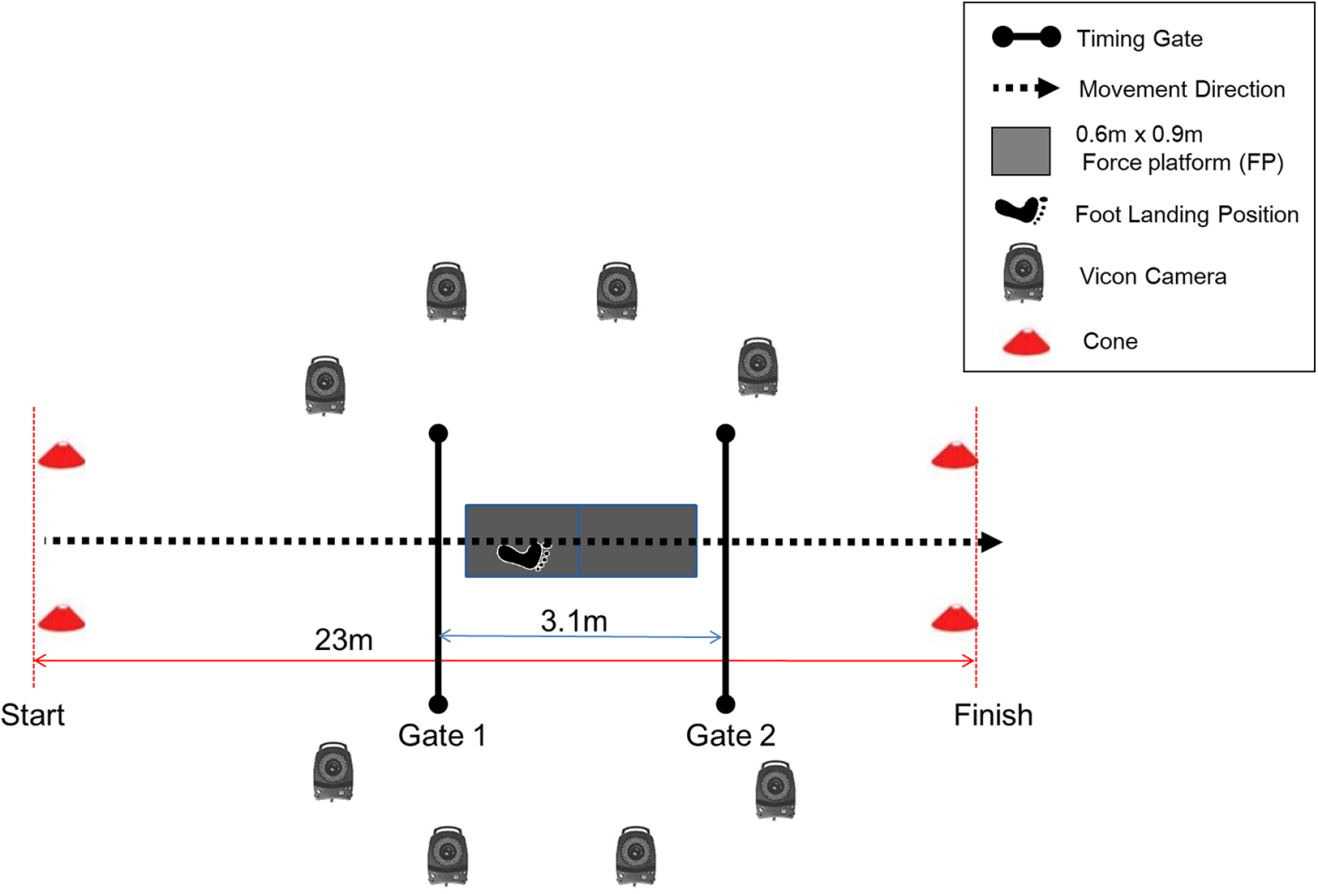
Experimental setup.

A tri-axial accelerometer (DTS 3D, 1,500 Hz; Noraxon, Scottsdale, AZ, USA) was securely affixed onto the antero-medial aspect of the proximal one-third of right tibia, with its vertical axis aligned along the tibia (Crowell & Davis, 2011). In addition, reflective markers were placed over participant’s right shoe with the method described in the previous running study done by Altman & Davis (2012). After a standardized warm-up protocol, participants were asked to perform five over-ground running trials with different footwear conditions (regular vs. better vs. best cushioned shoes) and at two different speeds (3.0 m/s vs. 6.0 m/s) on a flat, straight, 23-m long runway (Fig. 1). A successful trial was determined as a trial within 5% of the target speed and was done by placing timing gates (Smartspeed; Fusion Sport Inc., Burbank, CA, USA) before and after the force plate across the runway to determine target speed. The timing gate has been shown to have good test-retest reliability (Intraclass correlation coefficient = 0.88–0.97) for multiple test conditions (Green, Blake & Caulfield, 2011). A clean right footfall on the force plate (AMTI, Watertown, MA, USA) was needed for a successful trial. The force plate was mounted flush and located at the center of the 23-m runway. Test conditions were randomized using an online program (http://www.random.org). In order to ensure the participants were adapted to the specific testing conditions, they were allowed 3 min of treadmill running in the test condition and three familiarization trials prior to the data collection (Fellin et al., 2010). Two minutes of rest were provided between each test condition.

### Data acquisition and processing

**Figure 2:**
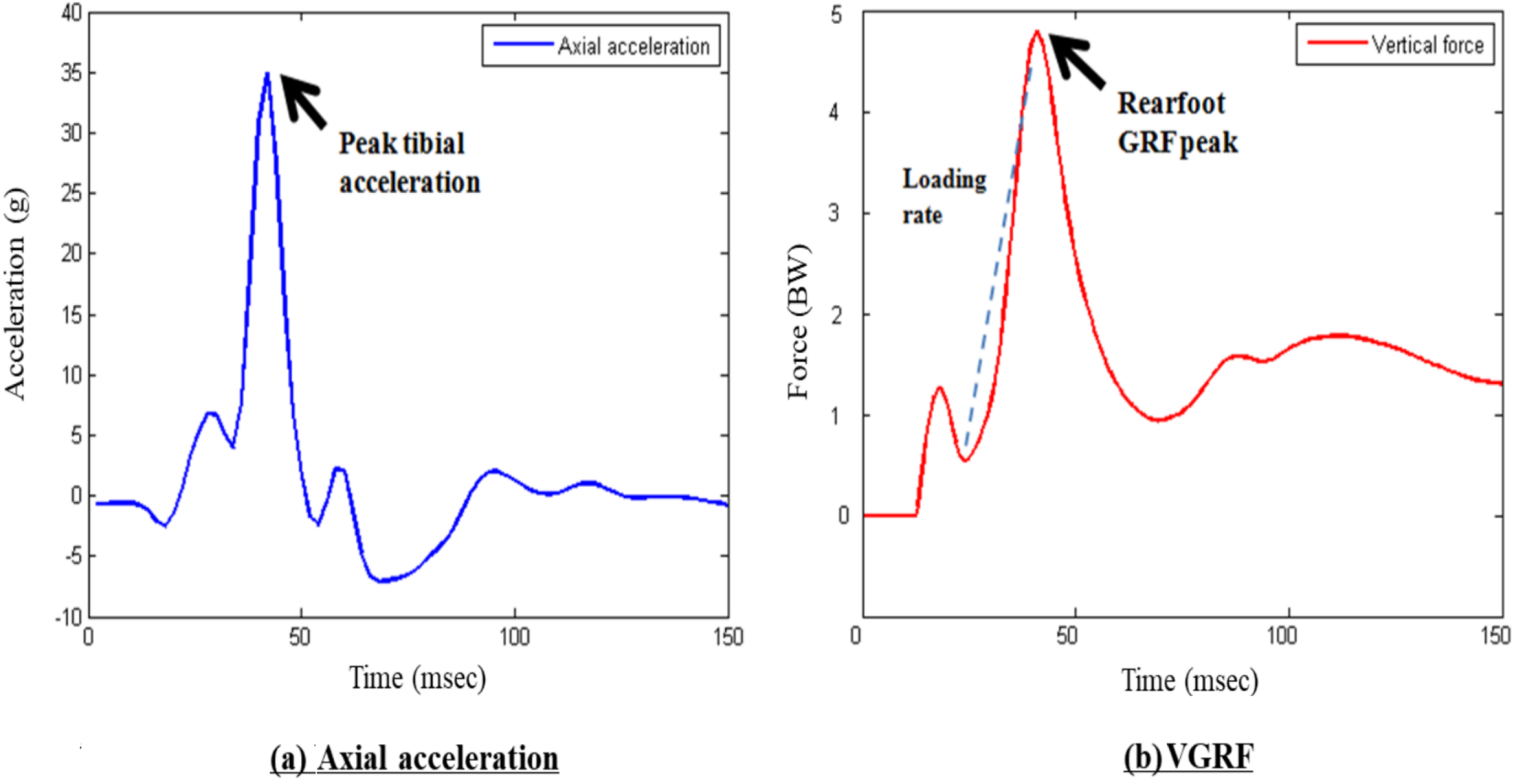
Sample curves of (A) tibial acceleration and (B) ground reaction force.

**Figure 3:**
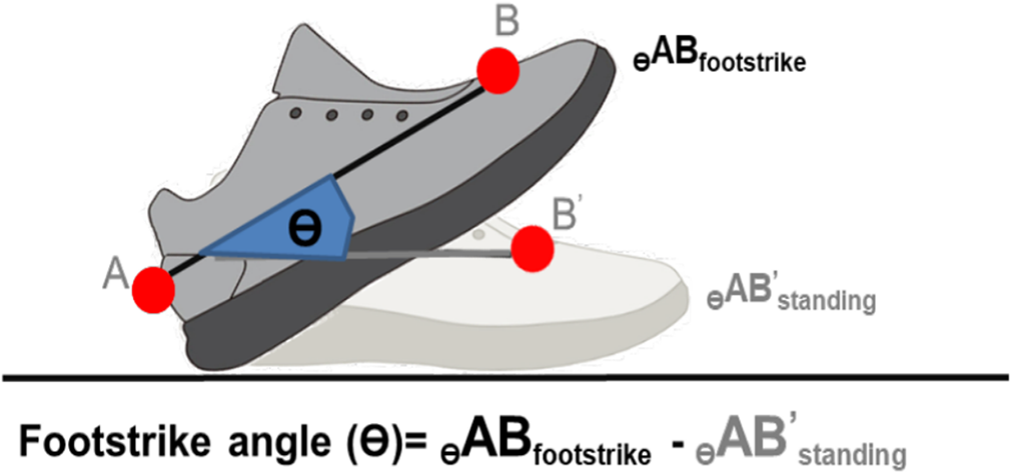
Definition of initial footstrike angle.

Tibial acceleration and vertical ground reaction force were recorded at 1,500 Hz. Motion data were captured using an eight-camera motion capturing system (Vicon T40s; Oxford Metrics, Oxford, UK) at 240 Hz. To synchronize all tibial acceleration, ground reaction forces and motion trajectory signals, each participant was asked to strike hard on the force platform with his right shoe before the data acquisition of each trial. Kinetics and kinematics data were filtered using a fourth order Butterworth low pass filter at 100 and 12 Hz, respectively and vertical ground reaction force data was body mass normalized for comparison across other studies. Tibial shock was defined as the maximum positive axial acceleration that occurred during the early stance phase of gait (Crowell & Davis, 2011) (Fig. 2A). Impact peak, vertical average loading rate (VALR) and vertical instantaneous loading rate (VILR) were calculated using the method described previously used by An, Rainbow & Cheung (2015, Fig. 2B). Impact peak is defined as the local maximum between foot strike and peak vertical force. VALR is the slope of the line from the 20% point to the 80% point of the impact peak (Blackmore, Willy & Creaby, 2016). VILR is the maximum slope of the vertical ground reaction force curve between the successive data points in the same region (20–80%). Initial footstrike angle was measured according to the method suggested by Altman & Davis (2012). The footstrike angles were defined as the difference between the angle of the foot at impact and the angle during standing. A footstrike angle of >8° indicated a rearfoot strike. A midfoot strike is determined if the footstrike angle lies between 8° and −1.6°; while foot a strike angle of <−1.6° indicates a forefoot strike (Fig. 3).

### Data analysis

All statistical analyses were performed using SPSS (Version 20.0; SPSS, Chicago, IL, USA). Prior to a 2 (speed) × 3 (footwear) repeated measures ANOVA, normality test (Shapiro–Wilk test) and Mauchly’s test of sphericity were performed the variables: tibial shock, VALR, VILR, and initial footstrike angle. Greenhouse–Geisser’s epsilon adjustment was used in all cases when Mauchly’s test indicated that the sphericity assumption had been violated. When significance was demonstrated in the ANOVA, pairwise comparisons were employed for any significant main effect. Effect size (η^2^) was interpreted as small (0 < η^2^ ≤ 0.02), medium (0.02 < η^2^ ≤ 0.09) and large (η^2^ ≥ 0.09). Estimated power (β) was interpreted as good when β was larger than 0.8. Level of significance was set at 0.05.

## Results

For vertical ground reaction force (Table 1), interactions between speed and shoe variables related to the vertical ground reaction force was not significant (*P* = 0.28). Participants experienced higher impact peaks, VALR, and VILR during the faster running speed (*P* < 0.001; η^2^ = 0.73; β = 1.00). Significant shoe effects were found on the impact loading variables (*P* = 0.03; η^2^ = 0.38; β = 0.92). Pairwise comparisons of shoe effect suggested that best-cushioning shoes resulted in a greater impact peak than regular-cushioning shoes (*P* = 0.04). The better-cushioning shoes presented lowest VALR and VILAR in comparison to the regular-cushioning shoes (*P* = 0.01) and best-cushioning shoes (*P* < 0.001).

**Table 1:** Tibial shock, vertical ground reaction force, and initial footstrike angle during each running condition in mean (standard deviation).

Speed		Shoe mechanical cushioning performance			Interaction			Speed			Shoe		
		Best-cushioning (Best)	Better-cushioning (Better)	Regular-cushioning (Regular)	*P*	η^2^	β	*P*	η^2^	β	*P*	η^2^	β
Tibial shock (g)	Slow	7.04(1.60)	6.36(2.04)	7.25(1.62)	0.49	0.09	0.15	**<0.001**	0.80	1.00	**0.03**	0.39	0.70
	Fast	10.86(1.95)	9.85(2.15)	10.11(2.45)									
Impact peak (BW)	Slow	2.05(0.30)	1.93(0.37)	1.95(0.34)	0.77	0.03	0.08	**<0.001**	0.87	1.00	**0.03**	0.38	0.92
	Fast	2.67(0.45)	2.57(0.40)	2.53(0.45)									
VALR (BW/s)	Slow	98.78(19.59)	83.82(25.89)	100.96(27.50)	0.30	0.15	0.24	**<0.001**	0.77	1.00	**<0.001**	0.68	1.00
	Fast	142.31(29.57)	121.93(32.20)	134.03(29.81)									
VILR (BW/s)	Slow	111.63(21.08)	100.14(29.53)	114.99(29.46)	0.28	0.15	0.25	**<0.001**	0.73	1.00	**<0.01**	0.61	0.98
	Fast	161.49(34.06)	140.80(35.53)	153.42(32.72)									
Footstrike angle (°)	Slow	21.44 (7.50)	22.81 (9.75)	21.82(0.22)	0.14	0.27	0.38	0.14	0.13	0.31	0.48	0.09	0.16
	Fast	21.18 (10.79)	18.18(14.99)	18.18(12.58)									

**Notes:**

BW, body weight; VALR, vertical average loading rate; VILR, vertical instantaneous loading rate; η^2^, partial eta squared; β, observed power.

Significant *P*-values (*P* < 0.05) are shown in bold.

For tibial shock (Table 1), no significant interaction effect between speed and shoe were found (*P* = 0.49). A significant main effect of speed indicated that greater tibial shock was observed at a faster running speed (*P* < 0.001; η^2^ = 0.80; β = 1.00). A significant effect with the shoe factor was found on tibial shock (*P* = 0.03; η^2^ = 0.39; β = 0.70). In the pairwise comparisons, best-cushioning shoes demonstrated significant greater tibial shock than better-cushioning shoes (*P* = 0.02).

Looking at initial footstrike angle (Table 1), the initial footstrike angle remained similar in all participants, regardless of the running speed and footwear conditions (*P* = 0.14). According to the cutoff angle suggested by Altman & Davis (2012), all of the participants landed with a rearfoot strike pattern during all test conditions.

## Discussion

The present study aimed to compare the tibial shock, impact peak, vertical loading rate, and initial footstrike angle when basketball players ran at different speeds wearing basketball shoes. As expected, basketball players experienced greater impact loading in terms of tibial shock, impact peak, VALR, and VILR at faster running speeds. The findings partly agree with a recent basketball study (Lam, Ng & Kong, 2017), which used in-shoe pressure soles to measure plantar loading. This study reported that basketball players running at faster speeds experienced significantly higher plantar loading (Lam, Ng & Kong, 2017). Similar to the general running population, the greater impact loading found in the results could be explained by the increased stride length and/or step frequency when running at faster pace (Mercer et al., 2002).

The relationship between mechanical and biomechanical shoe cushioning performance (i.e., mechanical impact scores vs. biomechanical impact loading) was in contrast with the original hypothesis. The results found that the impact load experienced by participants did not change systematically with changes in mechanical shoe cushioning properties. This is similar to previous studies where shoe cushioning performance did not systematically change with impact forces exerted on the participants during running (Nigg, 2010) or landing (Nin, Lam & Kong, 2016). This unsystematic relationship between cushioning and impact loading may be associated with participant’s body mass distribution which may result in bottomed out (sole material reaching very thin dimensions during impact) of shoe soles (Nin, Lam & Kong, 2016; Nikolaidis et al., 2015; Shorten & Mientjes, 2011). The another plausible explanation is that postural control, lower limb angles, leg stiffness or muscle activation may influence the effect of cushioning on impact forces (Paquette, Zhang & Baumgartner, 2013). The human body could unconsciously produce these movement patterns or initiate motor programs to avoid high impacts in different activities. For example, habitual rearfoot strike runners can change their natural movement pattern to a midfoot/forefoot striking pattern when running in a barefoot condition without priori instruction (Lieberman et al., 2010). Although more research is needed on these paradigms, it might be possible that basketball players (similar to runners) change their kinematics in response to shoe cushioning based on their individual “comfort filter” and “preferred movement path” paradigms, which suggested that a runner intuitively selects a comfortable shoe product using their own perception to allow for their preferred movement pattern (Nigg et al., 2015).

The initial footstrike pattern of the basketball players did not differ with respect to running speed and cushioning performance of the basketball shoes. This finding is in contrast with previous studies found in runners, which suggested that runners tend to land with non-rearfoot strike at faster running speed (Breine et al., 2014) or with less cushioning shoes (Moritz & Farley, 2004). This finding is particularly interesting since we tested similar running speed (3.0 and 6.0 m/s) as in the study by Breine and colleagues (3.2–6.2 m/s), which suggests the different motor execution patterns between basketball players and runners even at the same physical demand. One plausible explanation is that basketball players who are generally taller would adopt stride length strategy whereas runners with shorter legs would favor stride rate strategy for increasing running speed. Another plausible explanation is that apart from running, basketball players perform a large number of powerful jumps, accelerations and decelerations, lay-ups, and cutting in various movement directions (McClay et al., 1994; Ben Abdelkrim, El Fazaa & El Ati, 2007). These powerful movements require strong muscle strength in the lower limb. On the other hand, Paquette, Zhang & Baumgartner (2013) compared a minimal shoe model (FiveFingers KSO; Vibram, Concord, MA, USA) with a traditional running shoe model (Noveto; Adidas, Portland, OR, USA); while we selected usual basketball shoe models available in the market. The shoe cushioning performance of these basketball shoe models may not be comparable with the range covered by Paquette and colleagues.

Tibial stress fracture is one of the most common overuse injuries in basketball players. Shock absorption and impact attenuation are the primary considerations in sport footwear design to prevent impact-related injuries (Lake, 2000), and these are the most important features in basketball shoes (Brauner, Zwinzscher & Sterzing, 2012). The present study suggested that shoes with a medium impact score resulted in lower tibial shock and loading rates of impact loading than shoes with low or high impact score. Such findings may indicate a potential optimal band of shoe cushioning performance in lowering the risks of developing TSF among basketball players. Another possibility is that the players have better proprioception with shoes that have a lower cushioning score (Nigg et al., 2015). Considering the higher tibial shock and impact loading during the fast speed used in this study, basketball players who are recovering from TSF should avoid training regimens incorporated with sprinting, as a history of stress fractures is one risk factor for recurrent TSF (Larson et al., 2005; Hattori & Ito, 2015).

When interpreting the results, it is important to consider several limitations in the current study. Firstly, only male university basketball players were recruited and therefore the findings may not be generalized to female and professional basketball players before these populations are investigated. Secondly, the risk of TSF due to high running impact has been shown in running populations only, but not been well developed in basketball populations. In basketball, running contributes only a portion of the total impacts or loading, while other related movements such as change-of-direction running and jump landing might induce higher impacts with rapid deceleration. A total impacts or loading across typical movements that is encountered by a basketball player should be carried out before a viable conclusion can be made. Thirdly, the basketball shoe models used in this study were available in the market, which had different shoe constructions. Considering that cushioning performance incorporates all changes related to material viscoelasticity, midsole thickness and structures, studying shoe cushioning would allow comparing results across studies, especially with different footwear used. For this reason, future studies are warranted to investigate the impact loading at other isolated footwear structures (landing surface area, midsole hardness) as well as basketball related movements.

## Conclusion

Basketball players experience greater impact loading at faster running speeds, while impact loading did not show a significant difference between shoe cushioning properties. There may be an optimal band of shoe cushioning for better protection against TSF. Other footwear designs also have to be considered before better footwear recommendation can be made. Basketball players may remain rearfoot strike during running, regardless of running speed and footwear conditions. Our findings suggest future research on the differences between impact loading and foot strike patterns in runners versus basketball players, especially related to footwear development.

## Supplemental Information

10.7717/peerj.4753/supp-1 Supplemental Information 1 Total individual raw data (blinded).

## Additional Information and Declarations

### Data Availability

The following information was supplied regarding data availability: The raw data are provided as a Supplemental File.
